# Contemplative practices for healing historical trauma among BIPOC populations: a scoping review

**DOI:** 10.3389/fpubh.2026.1834789

**Published:** 2026-06-23

**Authors:** My Ngoc To, Ramona Beltrán, Keli King

**Affiliations:** 1 School of Social Work, Colorado State University, Fort Collins, CO, United States; 2 Graduate School of Social Work, University of Denver, Denver, CO, United States

**Keywords:** contemplative practice, historical trauma, Indigenous healing, intervention research, nature engagement, scoping review

## Abstract

**Introduction:**

Contemplative practices encompass a range of mental and physical exercises that cultivate awareness, steadiness, and compassion. While contemplative practices have been shown to treat trauma, their application toward historical trauma is limited. To address this gap, our scoping review aims to answer the following research questions: (1) What contemplative practices have been applied toward individual and collective healing of historical trauma, (2) what are the mechanisms by which contemplative practices can facilitate healing historical trauma, and (3) what research methods are used to develop, evaluate, and adapt contemplative practices for healing historical trauma?

**Methods:**

Rigorous search protocol were established to find English-language articles published after 1998 that were intervention studies incorporating contemplative practices to treat historical trauma or an outcome connected to historical trauma theory. Of 645 identified articles, 35 met eligibility criteria. Covidence software was used to extract general study information, sample characteristics, intervention details, and contemplative components. Thematic analysis was performed to identify mechanisms of healing across all articles.

**Results:**

Results showed a rising rate of publications within the last decade. Most articles were qualitative or mixed methods, applied community-engaged methods, and included Indigenous populations. Most interventions took place at a community (53%) or group level (36%), and more than half (58%) used contemplative practices as a core component. The most common practices were creative (56%), relational (53%), ritual (47%), and stillness (42%). Thematic analysis identified seven healing mechanisms: (1) Establishing Safety and Belonging, (2) Revitalizing Culture, (3) Increasing Well-being, (4) Processing Emotion, (5) Raising Consciousness, (6) Changing Narratives, and (7) Growing Empowerment.

**Discussion:**

This study provides a foundation to understand healing mechanisms within specific branches of contemplative practice in relation to historical trauma. Many culturally grounded contemplative practices support Indigenous well-being and should be recognized more explicitly in the contemplative science literature as effective, evidence-based approaches for improving public health in communities impacted by historical trauma. The fields of contemplative practice and historical trauma can be advanced by bridging Indigenous studies and BIPOC community practice with contemplative science, so long as this work continues to be carried out in deep partnership with and for BIPOC communities.

## 1 Introduction

Historical trauma denotes the collective trauma which occurs to a group of people who have been targeted with violence based on their shared identity (1–4). The impact of historical trauma on public health has been well documented among Black, Indigenous, and People of Color (BIPOC) communities across the world, including, but not limited to African Americans (5–8), Japanese Americans (9, 10), Cambodian Americans (11), Hmong Americans (12, 13), Armenians (14), Korean Americans (15), Mexican Americans (16), and many more. As the numerous negative impacts of historical trauma are increasingly documented, the broader arc of research and practice has shifted toward healing centered approaches that involve intervention studies.

Much of the present literature on historical trauma interventions has taken place among Indigenous peoples, whose foundational works address the healing of the “soul wound” (1, 17, 18) and later works study the impact of cultural revitalization on multiple facets of well-being (19–25). Many of these scholars conceptualize historical trauma as an embodied phenomenon in which collective and intergenerational harms influence biological stress systems, emotional regulation, and social outcomes (4, 26–28). This perspective aligns with growing public health evidence that such embodied impacts may be transmitted across generations through family communication (whether silence, fragmentation, or over-identification), ongoing exposure to structural violence, cultural and spiritual disruptions, and potential epigenetic changes. Understanding historical trauma in this way points to the potential promise of awareness-based and healing-centered interventions that directly engage these embodied patterns to promote healthy functioning, reduce internalized oppression, and support collective healing (29–32), particularly those incorporating contemplative and somatic practices.

Contemplative practices include a range of practices that focus on cultivating self-awareness, mindfulness, mental and physical steadiness, and compassion through mental training, which are often meditative in nature but can also include physical, arts based, or dialogical components (33, 34). Contemplative practices are rooted in numerous cultures across the globe, including, but not limited to religious and spiritual traditions that often overlap with Indigenous and diasporic lineages (35). The Center for Contemplative Mind in Society (33) outlines seven branches of contemplative practices, including stillness (sitting meditations), generative (savoring, sending thoughts of loving-kindness and compassion), creative (art-based expression, journaling), active (pilgrimage, activism), relational (deep listening, talking circles, storytelling), movement (walking meditation, mind-body practices), and ritual/cyclical practices (cultural ceremonies and traditions).

A robust body of literature has documented the suitable application of contemplative practices for healing a wide variety of traumas. Numerous studies have documented strategies to treat Post-Traumatic Stress Disorder and complex trauma with mindfulness-based interventions (36, 37), mind-body practices including yoga and movement-based practices (38, 39), and trauma-informed adaptation (40, 41). The scope of trauma-focused contemplative research has expanded in recent years to include diverse orientations and applications beyond clinical settings in which they had largely been secularized (42). Sister Dang Nghiem, for instance, has written a popular guide for healing personal trauma through a Buddhist practice lens that emphasizes compassionate mindfulness and body-based practices (43). In addition, contemplative practices have increasingly been directed toward healing collective trauma among BIPOC communities (32, 44–46). In these contexts, the focus of treatment extends beyond individually-focused trauma treatment to addressing internalized traumas that have collected over generations due to racialized oppression (32), and the modalities have extended to incorporate explicit cultural traditions such as African diasporic healing traditions (47).

More recently, contemplative practices are beginning to be explored as a promising modality for healing historical trauma, which is considered a related construct to racialized trauma (48). Yellowbird (49), for example, has been a leader on decolonial Indigenous contemplative frameworks, applying Indigenous approaches to mindfulness for fostering “neurodecolonization”. Numerous Black contemplative scholars and activists have also documented the utility of contemplative practices for healing racial trauma (32, 50, 51). While the application of contemplative practice to historical trauma has budded, a more comprehensive review of their application in this realm has yet to be executed.

To address the literature gap, this scoping review explores the range of contemplative practices which have been applied within interventions for communities affected by historical trauma in the last two decades. We intentionally uses the term *contemplative practice* given how mindfulness has become overused since its proliferation in mainstream society in the last two decades (52) and therefore is often misinterpreted as a medicalized and individual therapeutic option for reducing stress and regulating emotions (53). The term ‘contemplative practices' can be more inclusive by encompassing a broader range of practice modalities and culturally rooted forms of contemplative practice that may not necessarily fit within the scope of secular mindfulness. As such, the use of contemplative practices is more suitable for cross-cultural adaptation in efforts to heal historical trauma. This manuscript uses the term ‘contemplative practice' to broadly denote these seven branches of practice across multiple traditions, including culturally grounded contemplative traditions, secular mindfulness-based interventions, and broader community healing practices.

Following Arksey & O'Malley's (54) process, this scoping review aims to answer the following research questions: (1) What contemplative practices have been applied toward individual and collective healing of historical trauma, (2) what are the mechanisms by which contemplative practices can facilitate the healing of historical trauma, and (3) what research methods are used to develop, evaluate, and adapt contemplative practices for healing historical trauma?

## 2 Methods

### 2.1 Search criteria

Comprehensive search protocol was established to direct the search among the following databases: PsycINFO, Social Services Abstracts, Sociological Abstracts, Anthropology Plus, and ERIC. In addition, search results would also be gathered from Google Scholar (using the first 15 results pages) and relevant journals such as the *Journal of Traumatic Stress, Journal of Orthopsychiatry, Traumatology*, and *Transcultural Psychiatry, the Journal of Contemplative Inquiry*). Available reference lists on historical trauma such as those published by University of Minnesota on historical trauma research were also reviewed for relevant articles. The review utilized three primary search categories of historical trauma, intervention, and contemplative, resulting in the following Boolean Phrase:

*(historical trauma OR intergenerational trauma OR transgenerational trauma OR multi-generational trauma OR and generational trauma) AND (intervention OR healing OR growth) AND (mindfulness OR embodiment OR storytelling OR dance OR art OR movement OR ritual OR song OR ceremony OR awareness OR breath)*

The search terms were developed after numerous experimental searches. Terms within a category which did not result in numerical differences in search results terms (e.g., meditation, stillness practice, contemplative) were omitted from the Boolean phrase.

Articles were included if (1) they were an intervention study, defined in this review as the implementation of a treatment, program, or service to an individual, group or community with a description of outcomes, (2) the intervention incorporated contemplative practices either as the central component or supplement, (3) the intervention aimed to either directly treat historical trauma or another issue connected to historical trauma, such as addiction, incarceration, houselessness, or gender-based violence. Relevant literature included books and peer-reviewed quantitative, qualitative, and mixed methods articles that were written in English and published after 1998, following Danieli's (98) publication of the *International Handbook of Multigenerational Legacies of Trauma*, as well as Brave Heart's seminal work on healing historical trauma in the Lakota nation (1). There were no geographic restrictions on sources. Grey literature, which included dissertations, theses, and conference abstracts, were included if they met all criteria in the AACODS checklist (Authority, Accuracy, Coverage, Objectivity, Date, Significance) (55).

Articles were excluded if their target population was healthcare workers. Non-intervention studies, such as those encompassing autoethnography, theory, and review articles, were also excluded. Articles that did not explicitly apply a historical trauma theoretical framework to describe concepts, outcomes, and interventions were similarly excluded. Works implementing interventions that could not be categorized as contemplative practice or did not have rich enough description of the intervention to determine if it had contemplative components based on the definition of contemplative practice described earlier, were also excluded. The final search protocol was presented to an interdisciplinary team of peer reviewers for approval before proceeding with data collection.

### 2.2 Study selection

**Figure 1:**
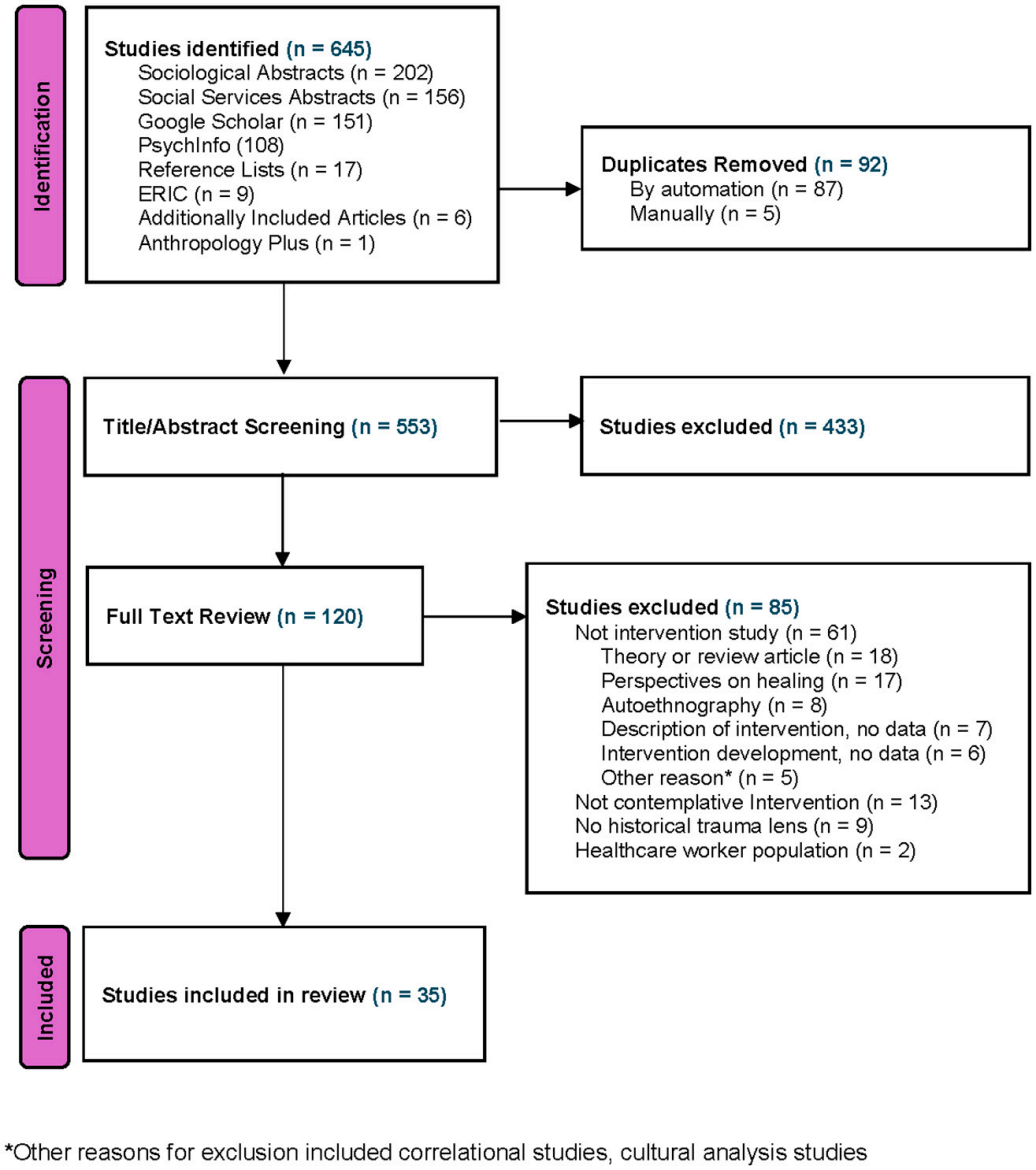
PRISMA chart.

The search resulted in 639 articles across all databases, with an additional six identified through reference lists, leading to a total of 645 retrieved articles (Figure 1). Article references were uploaded into Covidence, an online review management system and reviewed for duplicates before going through an initial review of titles and abstracts. Of the 120 articles included for full review, 35 met study criteria. Of these, grey literature included one PhD level dissertation (13). Because one article described two separate interventions (56), the final set of included articles represented 36 total interventions (Table 1). Due to funding and resource restrictions, screening, extraction, and analysis were performed solely by the first author. Rigor across these procedures was maintained through regular research meetings with a team of subject matter experts which included the second author. In place of resolving disagreements and conducting inter-rater reliability, the research meetings were utilized to review any pending inclusion decisions and other potential areas of bias during screening.

**Table 1:** Study information and intervention characteristics.

Citation	Region	Study design	Methodology	Community Engagement Level	*N*	Sample characteristics	Intervention name	Intervention description: [level] duration, structure	Contemplative parts: [grounded or supplemental], components
Atkinson (84)	Australia	One Arm Pilot	Qualitative: Assessment Report	Community Participation	60	Incarcerated adult Aboriginal women with history of complex trauma	Kunga (Young Women) Stopping Violence Program	[Group] 4 weeks (9–3 p.m. each weekday), twice a year; Culturally based program providing pre- and post-release support to incarcerated Aboriginal women	[Supplemental] Creative, Stillness, Relational
Baker (82)	Colombia	One Arm Pilot	Qualitative: Focus groups and observation	Participatory Action Research	13	Wayuu women (ages 18–54)	Embroidering Workshop	[Group] 3-days; Embroidering workshop where women created embroidered maps of their daily lives	[Grounded] Creative, Ritual
Barlow (63)	United States	Case Report – Single Event	Qualitative: Observation	Community Initiated^*b*^	55	African American college students (ages 18+)	Community Healing Network's Emotional Emancipation Circles	[Community] 1 day; Social support groups with learning modules dedicated to African culture, history and movements, and imperatives and ethics	[Grounded] Relational, Ritual
Barlowe and Thompson (74)	United States	Case Report – Single Event	Qualitative: Observation	Community Participation^*a*^	–^*^	Native American adult males in prison system	Healing Conference	[Community] 5-days; Conference held at Maximum Security Oregon State Prison that provided psychoeducation and a full day work shop on Traditional Ceremonial Recovery Strategies	[Supplemental] Ritual
Barudin (69)	Canada	Case Report - Retrospective	Qualitative: Observation	Community Participation	8	Inuit female youth (ages 13-17) with history of child maltreatment under the care of youth protection services	Trauma-Informed yoga.	[Group] 12 weeks (60-min weekly sessions); Trauma-informed yoga group at an urban residential facility	[Grounded] Stillness, Creative, Relational, Movement
Beltrán and Begun (20)	Aotearoa (New Zealand)	One Arm Pilot	Qualitative: In-depth interviews	CBPR	6	Maori adults (ages 20–45)	Aotearoa Digital Storytelling as Indigenous Media Project	[Group] 4-days; Intensive workshop where participants engaged in a 7-step digital storytelling process that combined social/relational, literacy, and technological skill development	[Grounded] Creative, Relational
Black et al. (21)	Australia	Case Report – Ongoing Program Evaluation	Qualitative: Semi-structured interviews and written responses	CBPR^*c*^	3	Aboriginal women (early to mid-twenties)	Narran Yana (Spirit Journey) art collective	[Community] Ongoing duration; Art collective designed by youth, for youth as an innovative cultural strengthening program	[Grounded] Creative, Ritual
Brave Heart (1)	United States	One Arm Pilot with long-term follow-up	Quantitative: Surveys	CBPR^*c*^	45	Lakota adult women	None stated	[Group] 4 days; Culturally congruent group program offering psychoeducation on Lakota trauma and unresolved grief, small group exercises, and ceremony	[Supplemental] Ritual
Budowle (78)	United States	One Arm Pilot	Qualitative: Focus groups, sovereign storytelling interviews	CBPR^*c*^	22	Eastern Shoshone and Northern Arapaho adults	Growing Resilience	[Community] 2 years; Community-based participatory gardening pilot study	[Grounded] Nature Engagement, Relational
Dudgeon et al. (22)	Australia	One Arm Pilot	Mixed Methods: Post-program evaluation form, semi-structured individual interviews	Community Driven^*a*^	49	Aboriginal adults (ages 18+)	Cultural, Social, and Emotional Wellbeing	[Community] 12 weeks (6.5 h weekly sessions); Aboriginal-led community initiative that aims to strengthen individua Social Emotional Well-being and promote family and community well-being	[Grounded] Stillness, Creative, Relational, Movement, Ritual, Nature Engagement
Gone (80)	Canada	One Arm Pilot	Qualitative- semi structured interviews with participants and staff	CBPR^*a*^	19	Native American adults	Counseling program at “the lodge”	[Community] 10 weeks, (Four 2–4 h sessions/wk); Outpatient counseling program within a First Nation community treatment setting that integrates cultural components	[Supplemental] Stillness, Creative, Relational, Ritual, Movement
Hargons et al. (45)	United States	Randomized Experimental Pilot	Mixed Methods: Heart rate, semi structured interviews	N/A	26	African-American college students(18–30)	Black Lives Matter Meditation for Healing Racial Trauma	[Individual] 17-min; Online recorded guided meditation designed for race-based stress	[Grounded] Stillness, Generative
Heilbron and Guttman (83)	Canada	One Arm Pilot	Qualitative: Open-ended evaluation forms and group recordings	CBPR^*a*^	5	Ojibway, First Nations and non-aboriginal women (age mid 30s - mid 40s) with history of child sexual abuse	Healing Circle	[Group] 10 weeks (2 h weekly sessions); Healing circles within counseling group at Native Social Services Branch	[Supplemental] Relational, Ritual
Hughes (46)	United States	One Arm Pilot	Quantitative: Evaluation survey	Community Participation^*b*^	10	African-American adults	R.E.S.T.: A Practice for the Tired and Weary	[Group] 5-weeks; Online meditation group on historical experiences of African Americans	[Grounded] Stillness
Johnson-Jennings, Koushik et al. (70)	United States	One Arm Pilot	Mixed Methods: Surveys, focus groups, interviews with key informants	CBPR^*c*^	7	Native American youth (ages 5-11) from mostly Anishinabe tribes	Ode,imin Giizis *(Strawberry moon, or sixth moon)*	[Community] 6 months (3-4 times a week); Cultural, strengths-based urban rooftop gardening pilot implemented within housing center	[Grounded] Movement, Nature Engagement
Johnson-Jennings et al. (23)	United States	One Arm Pilot	Mixed Methods: Body composition, interviews, semi-structured focus groups	CBPR	20	Huoma women (ages 18-45)	Returning to Our Roots	[Community] 7 days, 126 miles; Outdoor, land-based healing pilot retracing the forced migration of Huoma ancestors	[Grounded] Stillness, Active, Nature Engagement, Movement
Kinsey (81)	United States	Case Report – Ongoing Program Evaluation	Mixed Methods: In-depth interviews and participant observation	CBPR^*c*^	13	Squamish adult tribal members	Healthy and Whole	[Community] Unclear duration; Culturally adapted DBT meant to provide mental health treatment that recognizes historical origins of tribal mental health problems	[Supplemental] Stillness, Generative, Active, Ritual
Ko (65)	United States	Case Report – Individual	Qualitative: Observation	N/A^*c*^	1	40-year old Korean-American woman with history of intimate partner violence and *haan*	*Salpuri* (Korean Scarf Dance)	[Individual] 10 weeks (60 min sessions); Weekly expressive therapy sessions incorporating traditional Korean scarf dance	[Grounded] Movement
Kohrt et al. (71)	United States	Case Report - Individual	Mixed Methods: Surveys, DBT daily diary card, observation	N/A	1	14-year old Navajo woman with Major Depressive Disorder with Psychotic Features	Dialectical Behavioral Therapy (DBT)	[Individual] 3 months; 7 weeks inpatient care at acute center for children (individual therapy 2x/week, family therapy 1x/week, and group DBT 2x/week) followed by 1 month outpatient care	[Supplemental] Stillness, Creative, Ritual
Le and Gobert (72)	United States	One Arm Pilot	Mixed Methods: Surveys, facilitator written reflections, open-ended interviews with participants, facilitators, and administrators	CBPR^*c*^	8	Salish and Kootenai Tribal youth and young adult members (ages 15-20) of the Flathead Reservation in Rural Northwestern Montana	Restoring the Native American Spirit	[Group] 9 week class (4 sessions/wk, 45-min per session); Program adapted from Mind Body Awareness Project conducted in council styles as part of larger Circle of Trust Suicide Prevention Program	[Grounded] Stillness, Generative, Relational, Ritual
Lewis et al. (73)	United States	Case Report – Retrospective	Qualitative: Focus groups and some written comments	Community Driven^*b*^	23	Cherokee youth and young adults (ages 16-24)	Remember the Removal	[Community] 6.5 months, 950 miles; Cherokee nation-building program culminating in a 3-week bike ride along the Trail of Tears	[Supplemental] Creative, Active, Movement, Ritual, Relational, Nature Engagement
Liem (15)	United States	Case Report – Single Event	Qualitative: Observation	N/A^*c*^	–^*^	General public	Still Present Pasts: Korean Americans and the “Forgotten War” Art Exhibit	[Community] Varying duration; Multimedia art exhibit featuring installation and performance art, documentary film, archival photographs, and interactive elements inspired by and embodying oral history voice	[Grounded] Creative
Marsh et al. (85)	Canada	One Arm Pilot	Mixed Methods: End-of-session surveys, focus groups, semi-structured interviews with participants and facilitators	CBPR^*c*^	24	Aboriginal adults (Ojibway, Cree, and Metis) in Northern Ontario (ages 24–68) who self-identify as having experience Intergenerational Trauma and Substance Use Disorder	Indigenous Healing and Seeking Safety	[Community] 13 weeks (2-h sessions twice a week); Adaptation of mainstream Seeking Safety program with Indigenous frameworks, methodologies, and approaches to treat Intergenerational Trauma and Substance Use Disorder	[Supplemental] Stillness, Creative, Ritual, Generative, Relational
Nelson et al. (75)	United States	Case Report – Ongoing Program Evaluation	Mixed Methods: Surveys, open-ended interviews with patients, providers, and management and traditional health providers	N/A^*c*^	31	Native American/Indigenous adults (ages 21+)	Harm-Reduction Talking Circles	[Group] 8 weeks; Weekly Talking Circles focused on harm-reduction for people with Alcohol Use Disorder	[Supplemental] Relational, Ritual
Niwenshuti (64)	Rwanda	Case Report – Single Event	Mixed Methods: In-depth interviews, focus groups, observation visits	N/A	40	Rwandan youth (ages 10–18)	Cultural Group	[Community] 6-months; School-based cultural group where youth applied dance to communicate experienced and emotions around Rwandan Genocide	[Grounded] Creative, Movement
Pells et al. (56)	Rwanda	Case Report – Single Event	Qualitative: Observation	N/A	20	Rwandan youth and young adults (ages 12-20)	Connecting Memories	[Community] 3 days; Workshop designed to help youth link current suffering to past events through recollection	[Grounded] Creative, Relational
Pells et al. (56)	Rwanda	Case Report – Single Event	Qualitative: Focus groups	N/A	10	Rwandan youth (specific age not stated)	Mobile Arts for Peace and Mobile Filmmaking	[Community] 3 days; Workshop on making films about peace-building using mobile phones, guided by Rwandan film maker	[Grounded] Creative
Ramirez (68)	United States	Case Report – Single Event	Qualitative: Observation	N/A	–^*^	General public	American Indian Holocaust Exhibit (1996)	[Community] Varying duration; Interactive art exhibit intended to counter cultural/colonial sanctions of violence against Native American women	[Supplemental] Creative, Ritual
Rebolledo (66)	Bangladesh	One Arm Pilot	Qualitative: Focus groups	CBPR^*c*^	850	Youth and adult Rohingya refugees in Bangladesh (ages 12–70)	Healing Ceremonies Program	[Community] 6 weeks (3 biweekly 60-min sessions); Program designed to provide a space for refugees to reconnect with their memory heal collectively	[Grounded] Creative, Ritual, Stillness
Schultz et al. (24)	United States	One Arm Pilot	Qualitative: In-depth interviews and focus groups	CBPR^*c*^	6^∧^	Tribally enrolled Choctaw women (ages 21-46)	The Walk	[Group] 9-days, 254 miles; Outdoor, land-based healing pilot that retraced forced migration of Choctaw across Arkansas	[Grounded] Stillness, Active, Movement, Nature Engagement
Thomas and Bellefeuille (77)	Canada	One Arm Pilot	Qualitative: Conversational-style interviews and focus groups	N/A	6	Aboriginal adults (ages 25–65) from Winnipeg, Manitoba with prior boarding school experience	Cross cultural mental health program	[Group] 12 weeks (8 total 3-h group sessions); Mental health program combining Aboriginal healing circles with self-awareness psychotherapy technique of ‘focusing'	[Supplemental] Stillness, Generative, Relational
Victor (79)	Canada	Case Report – Ongoing Program Evaluation	Qualitative: Semi-structured interviews with program participants and key informants	Community Participation^*a*^	24	Niitsitapi adults (ages 18+) who are unhoused	*I'taamohkanoohsin* (Everyone Comes Together)	[Community] Ongoing bi-weekly 3–4 h events; Program that raises a Tipi in a park to increase access to Blackfoot cultural activities for unhoused Niitsitapi	[Supplemental] Creative, Relational, Nature Engagement
Wilmott et al. (76)	Australia	Case Report – Ongoing Program Evaluation	Qualitative: Interviews and evaluations	CBPR	23	Aboriginal adults (ages 18+) with history related to Stolen Generation	Healing Camp	[Community] 4-day; Residential healing camp focused on well-being of First Nations people and their communities offering both Indigenous and Western approaches to healing	[Grounded] Creative, Relational, Movement, Ritual
Wimbish-Cirilo (62)	United States	Two-condition Quasi-experimental Study	Quantitative: Surveys	CBPR^*a*^	100	Urban Native American youth (ages 10–12) from Native American youth community center	Urban Talking Circle	[Group] 10-weeks (45-min sessions); Weekly talking circles intended to reduce interest in drug use among Urban American Native Youth	[Grounded] Relational
Womersly and Arikut-Treece (67)	Kurdistan Region of Iraq	Case Report – Ongoing Program Evaluation	Mixed Methods: Surveys, semi-structured focus groups, in-depth interviews with project team members	N/A	200	Adult Yezidi women who returned from Islamic State (ISIS) Captivity or had been displaced by attacks	Free Yezidi's Mental Health Intervention	[Individual, Group, and Community] Multi-level mental health program providing psychoeducation, group EMDR, and community workshops	[Supplemental] Stillness, Creative, Generative, Movement, Relational, Nature Engagement
Xiong (13)	United States	One Arm Pilot	Mixed Methods: Semi-structured interviews, evaluation form	Community Participation^*c*^	9	Hmong women (ages 15-65)	Hmong Women's Conference on Historical Trauma	[Community] 4 days; 2-day training following by 2-day conference consisting of psychoeducation on Hmong history and historical trauma	[Supplemental] Creative, Relational

^*^ Sample size not stated.

^∧^ Represents subsample of larger intergenerational group with youth participants ages 12–16.

^*a*^Someone from the research team has worked extensively with the community.

^*b*^Someone from the research team is part of the community.

^*c*^Someone from the research team is from, and has worked extensively with the community.

### 2.3 Data extraction

Full article review included data extraction on general study information as well as information relevant to the research questions. General study information included the year of publication, country where the research took place, study design, and methodology, including the depth of community engagement (57). Information on study sample included sample size and sample characteristics such as age, gender, and background. Unless otherwise noted, adult populations were assumed to be ages 18 and older. Data about interventions included the intervention name and a brief description of the intervention, including the intervention level – individual, group, or community. Intervention details also included the duration of the intervention and aims of the intervention.

Contemplative components of the intervention were extracted on whether the intervention was mainly established in contemplative practice (Grounded) or applied contemplative practices as a supplement to other components (Supplemental). Note was taken of which parts of the intervention matched the general categories of contemplative practice as described within the Contemplative Tree: Stillness, Generative, Creative, Active, Relational, Movement and Ritual Finally, articles were extracted for mechanisms of healing which occurred, specifically related to the role of contemplative practices on outcomes. The article search and review was performed by the first author with assistance from the third author on the aspect of depth of community engagement. The findings and descriptions were validated and discussed with the second author.

Aside from determining if the study met the requirements to be an intervention study, a formal quality assessment was not conducted. Given that quality assessments often prioritize experimental and randomized designs, integrating one would have potentially restricted community-based and pilot studies conducted with smaller samples. As such, a formal quality assessment was not performed into order to prioritize the scoping review's primary goal of gaining a comprehensive understanding of the topic, identify research gaps, and consider future directions. The rigor of included studies is nevertheless reviewed in the discussion section.

### 2.4 Thematic analysis

Mechanisms of healing across qualitative and mixed methods articles were identified by following Braun and Clarke's steps for reflexive thematic analysis (58). The initial step of familiarization took place via data immersion during full text review (59). Initial codes based on healing were then generated based on healing narratives discussed within each article. Healing was defined as instances of symptom reduction, empowerment, and other positive outcomes that were attributed to the intervention. Subsequently, text from the results and discussions sections of qualitative and mixed methods articles were extracted and uploaded into Dedoose, a qualitative analytic software, as transcripts for analysis. Transcripts were then coded using the initial codes of healing described above. Initial codes were consolidated into a codebook that was then used to refine code application across all articles (60). Themes of healing mechanisms were then identified across the data and discussed in a research team meeting for validity and consensus.

## 3 Results

The results of this scoping review are articulated based on Levac et al.'s (61) recommendations to present Arksey and O'Malleys (54) charted data in three sections: (1) Reporting article descriptives, (2) reporting results most pertinent to guiding research questions, and (3) discussing the meanings of the findings as they relate to the overall study purpose, including implications for future research, practice, and policy.

### 3.1 Article descriptives

#### 3.1.1 Geographic regions and publication years

Out of the 35 articles reviewed (Table 1), 19 (54%) were from the United States, six (17%) from Canada, and four (11%) from Australia. Two studies were from Rwanda, and one study each was done in New Zealand, Bangladesh, Colombia, and the Kurdistan Region of Iraq. Most articles were relatively recent. Five articles (14%) were published before 2010, with the earliest published in 2000, and another five articles (14%) published from 2010–2014. The next period from 2015–2019 showed an increase in publications, with 12 published articles (34%). The trend continued with 13 articles (37%) published from 2020 onward, demonstrating a trend of increasing application and exploration in the literature.

#### 3.1.2 Study designs and methodologies

Almost half of the studies were one-arm non-experimental pilot studies (*n* = 17, 49%), one of which included long-term follow-up (1). The second most common study design was case reports (*n* = 16, 43%) that included analysis of single-occurring events or programs (*n* = 6), evaluations of ongoing programs (*n* = 6), individual case studies (*n* = 2), and retrospective case reports on programs (*n* = 2). Only two studies employed two-arm designs (45, 62). More than half of the studies utilized qualitative methods (*n* = 20, 57%), about a third used mixed methods (*n* = 12, 34%), and a small fraction applied quantitative methods (*n* = 3, 9%). More than two thirds of the studies (*n* = 25, 71%) incorporated a range of community engaged research methods. These included six community participation studies, one Participatory Action Research (PAR) project, one community-initiated study, 15 Community-Based Participatory Research (CBPR) projects, and two community-driven initiatives.

#### 3.1.3 Sample characteristics

The majority of studies included Indigenous populations in their sample (*n* = 24, 69%). Tribal communities represented include the Choctaw, Lakota, Eastern Shoshoone, Northern Arapahoe, Huoma, Squamish, Navajo, and Cherokee in the United States; Niitsitapi, Ojibway, Cree, Metis, First Nations, Inuit, Maori, Yezidi, Aboriginal, and Wayuu. Three studies had samples representing African Americans (45, 46, 63), two of which focused on college students. Another two studies looked at a Rwandan sample (56, 64). Two studies worked with immigrant populations: a group of Hmong American women (13) and a case study following a Korean American woman (65). Two studies focused its sample on international refugees (66, 67), and two studies were open to the general public (15, 68).

Ages represented among samples ranged largely. The majority of studies (*n* = 23, 66%) focused on adult samples, with ages ranging from 18 to 70, though some studies did not give indication of ages and simply referenced “adult” populations. Five studies (17%) focused solely on youth (62, 64, 69–71). Five studies included both youth and adults in their sample, with three incorporating youth and young adults (56, 72, 73) and two incorporating an intergenerational sample (13, 66). One study published data of an adult subsample from a broader intergenerational sample in order to focus on the experiences of adult Choctaw tribal members (24). The two studies which gathered data from a general population did not report ages of their sample. A significant number of studies focused on solely female populations in their sample (*n* = 11, 31%) while one study had a solely male sample of Native American men within a prison system (74).

Samples presented people with a broad range of histories. Some samples required participants to have specific clinical conditions, symptoms, or self-identified lived experiences for inclusion. These included having histories of intergenerational trauma, substance use disorder, houselessness, incarceration, involvement with child welfare systems, or, particularly among female samples, sexual violence. Only two studies applied a DSM-V diagnosis in inclusion criteria (71, 75). Some studies looked at historical experiences, such as being affected by the Rwandan genocide (64), having a history related to the Stolen Generation (76), or boarding school experience (77). The majority of studies recruited participants from the general community or community events and intentionally did not apply strict inclusion criteria in order to make the intervention as inclusive and welcoming as possible. The breadth of variation in sample backgrounds can be reviewed in detail within Table 1.

### 3.2 Intervention characteristics

#### 3.2.1 Intervention level

Most of the interventions took place at a community level (*n* = 19, 53%). These included art exhibitions (15, 68), conferences (13, 63, 74), healing camps (76), gardening pilots (70, 78), art collectives (21), an extended Cherokee nation building program (73), school-based groups (64), the IĆtaamohkanoohsin program which erected tipis for unhoused Indigenous community members (79), and mental health groups based within community programs based on reservations (80, 81).

Group level interventions (*n* = 13, 36%) included those offered through existing social services organizations, workshops (20, 82), and online meditation group (46), and out-door land-based healing pilots (23, 24). Only three interventions (8%) took place at an individual level (45, 65, 71), and one intervention took place at all three levels (67).

#### 3.2.2 Intervention duration

The duration of interventions ranged from 17 min to 6.5 months. Most of the interventions were offered as weekly programs, ranging from 4 to 13 weeks with one to four sessions a week. The length of each session varied from 45 min to 6 h per session. A number of interventions provided intensives such as workshops and conferences lasting on average 3–4 days, with some as short as one day (63) and some much longer as in the case of the Remember the Removal program which culminated in a 3-week long bike ride through the Trail of Tears (73). Some interventions developed by community members such as the Narran Yarra Art Collective (21) and the I'taamohkanoohsin program (79) were ongoing in nature, and the art exhibitions mentioned did not state a definite duration of the art exhibit (15, 68).

#### 3.2.3 Intervention aims

The diversity of intervention durations and sample characteristics is reflected in the assortment of aims. Interventions located within social service agencies and mental health organizations sought to address issues of houselessness, substance use, suicide, childhood obesity resulting from systemic food colonization, incarceration, complex trauma, and promoting individual and community social emotional wellbeing. Two interventions focused specifically on reducing symptoms related to race-based stress (45, 46). A number of interventions focused on storytelling, either by having participants recount their personal narratives (20, 56, 82), embroidery (82), film-making (56) or by countering broader societal narratives of silence (13, 15, 56, 64, 68).

Many interventions, either instead of or in addition to these objectives, focused on revitalizing culture. This is exemplified by the Community Emancipation Circles dedicated to immersing African-American college students in African culture, history, and movements (63), the Narrun Yarra cultural strengthening collective arts collective, (21), an Aboriginal-led community initiative aimed at strengthening individual and collective well-being (22), the Cherokee nation-building Remember the Removal program (73), and pilgrimages (23, 24). Other interventions increased community access to cultural knowledges and traditions by hosting cultural events that could counteract “spiritual homelessness” (79), providing a one-day workshop of traditional healing ceremonies at a conference within a maximum security prison (74), offering a space for Rohingyan refugees to reconnect with their ceremonies (66), and having both Indigenous and Western healing approaches at healing camps for First Nations people (76).

Other studies increased cultural access by adapting existing programs according to Indigenous frameworks and practices. This was achieved through integrating talking circles into established modalities (62, 72, 75, 77, 83); bringing indigenous traditions into a trauma-informed yoga group (69), integrating Korean scarf dance into expressive arts therapy sessions (65), and offering culturally congruent psychoeducation groups to Lakota tribal members which included ceremony (1).

At times, interventions were created by infusing existing programs with Indigenous frameworks, including the Counseling Program at the Lodge, which integrated cultural components into a First Nations community clinic (80), the Kunga Stopping Violence Program which offered culturally-based programming for incarcerated Aboriginal women (84), Seeking Safety which applied Indigenous frameworks to the mainstream Seeking Safety Program (85), Healthy and Whole which culturally adapted DBT for the Squamish tribe (81), and Restoring the Native American Spirit which adapted the Mind Body Awareness Project to include council circles (72).

#### 3.2.4 Contemplative components

Most interventions utilized contemplative practices as the core of the intervention (*n* = 21, 58%), while the rest used contemplative practices as a supplement (Table 1). The summary of contemplative practices utilized across interventions can be found in Table 2. Across all the interventions, creative practices were the most common (*n* = 20, 56%), encompassing numerous forms of visual art, expressive arts, crafting, and writing. The next most common contemplative practice was relational (*n* = 19, 53%), which included talking circles, dialogue, storytelling, and active listening. Almost half of the interventions (*n* = 17, 47%) had ritual components including prayers, ceremonies, traditional medicines, and traditions. Fifteen of the studies (42%) featured stillness practices which included various forms of meditation, mindfulness, and reflective activities. Eleven interventions (31%) included movement practices such as yoga, walking, canoeing, stretching, biking, and dance. Seven of the interventions (19%) were forms of nature engagement including activities such as gardening and hunting. While this was not an original category of contemplative practices, a unique category was made due to the salience of nature engagement among the interventions. Six of the interventions (17%) were generative practices such as loving-kindness meditation, self-compassion meditation, affirmations, cultivating compassing and empathy, and acceptance Finally, the least number of interventions (*n* = 4, 11%) incorporated active practices such as pilgrimage and activism. The majority of interventions implemented multiple categories of contemplative practice at once.

**Table 2:** Contemplative practices by category.

Contemplative branch	Practices
Active	•Activism (81) •Pilgrimage (23, 24, 73)
Creative	•Beading (69, 79) •Contemplative arts (68) •Drumming (79, 85) •Embroidery (82) •Film-making (15, 20, 21, 56) •Games (67) •Journaling (13, 20, 71, 73) •Letter writing/burning (80) •Music (66, 84, 85) •Performance/theater (15, 56, 64) •Photography (15, 56, 68) •Poetry (64, 84) •Proverbs (56) •Scrapbooking (84) •Sewing (67) •Singing (21, 22, 64, 67, 76, 79, 85) •Visual Art-Drawing and Painting (56, 66–68, 71, 76, 84) •Yarning (22, 84)
Generative practices	•Acceptance (81) •Affirmations (46) •Commemorative activities (67) •Cultivating compassion and empathy (72) •Elder teachings (85) •Loving kindness meditation (45) •Self-compassion meditation (77)
Stillness	•Body scan (84, 85) •Centering/grounding practice (28, 67, 69) •*Dadirri* meditation (84) •Guided imagery/visualization (80) •Meditation (22, 24, 45, 46, 69, 80) •Mindfulness ((22, 45, 70–72, 81)) •Practicing faith (66) •Contemplative reflections (23)
Movement practices	•Biking (73) •Canoeing (23) •Cooking (23) •Dancing (22, 64, 65, 67, 76, 80) •Stretching (23) •Walking (23, 24, 76) •Yoga (23, 69)
Relational	•Active listening (20, 63, 72) •Collective sharing (67) •Connection (72, 73, 76, 79, 80, 83) •Cultural connectedness (73) •Dialogue (13, 20, 63) •Powwow (79, 80) •Sharing/talking circles (56, 62, 69, 75–80, 83–85) •Storytelling (13, 20, 22, 56, 76, 79, 85)
Ritual	•Ceremonies (1, 22, 66, 72–74, 76, 80–83, 85) •Fasting (80) •Giving thanks to ancestors (63) •Navajo healing traditions (71) •Prayer (66, 72, 75, 80, 85) •Sacred bundle (85) •Smudging (72, 75, 80, 85) •Spiritual blessing (68) •Sweat lodge (80, 85) •Tobacco offerings (80, 85) •Traditional crafts: Possum-skin cloak making (21), Tjanpi weaving (76) •Traditional herbal healing (75, 85)
Nature engagement	•Fishing^*^ (79) •Gardening^*^ (67, 70, 78) •Hunting with Elders^*^ (22) •Land-based education (73) •Land-based healing (23) •Traditional activities (79) •Visiting sacred sites (79) •Wilderness experience^*^ (24)

^*^These activities are also considered Movement contemplative practices but are categorized as nature engagement due to the explicit focus on connecting with nature.

### 3.3 Mechanisms of healing

Thematic textual analysis on the healing mechanisms described across all articles resulted in identification of seven main healing mechanisms: (1) Establishing Safety and Belonging, (2) Revitalizing Culture, (3) Increasing Well-being, (4) Processing Emotion, (5) Raising Consciousness, (6) Changing Narratives, and (7) Growing Empowerment.

#### 3.3.1 Establishing safety

An essential factor for healing was the creation of a safe group space marked by openness, cohesion, vulnerability, acceptance, and non-judgement which fostered a sense of belonging (13, 15, 20–22, 66, 67, 69, 74, 76, 77, 79, 83). Participants shared that the level of acceptance they felt in two substance use programs that allowed people to come inebriated helped them accept themselves more (75, 79). Oftentimes, the group container felt like a sacred space, which contributed to a sense of safety because “you can't harm people in a ceremony” [(75), p. 40). Social connectedness through strengthened family and community relationships played a significant role in increasing a sense of belonging and respect, which supported healing (67, 73, 75, 77).

#### 3.3.2 Revitalizing culture

The cultural strengthening components of the interventions where participants reclaimed lost skills, knowledge, and appreciation for past generations had powerful roles in facilitating the healing process, especially when there were extensive histories of cultural dislocation and dehumanization (56, 62, 69, 73, 78, 83). Cultural revitalization led to increased pride and confidence in one's identity and cultural heritage (1, 21, 23, 74, 79, 80). It also decreased sense of isolation due to a sense of connection with other people and an understanding of the sacred interrelationship that one has with the world (62, 85). Learning culturally relevant coping skills led to a reduction in substance use (62), and re-establishing relationships to ancestral lands and food allowed participants to nourish a sense of attachment to country (22) and initiate behavior change (23).

#### 3.3.3 Increasing well-being

Healing was also accomplished through improvement in socioemotional well-being. Participation in healing ceremonies played a significant role in increasing sense of well-being. During ceremony, participants could reconnect with sources of strength such as music (66), gain a sense of calm (22), nurture their spirit (79), and gain self-esteem, efficacy, and trauma mastery (1, 80). Practices of stillness, such as tuning into nature, finding inner silence, and open awareness increased wellbeing by enhancing participants' abilities to emotionally self-regulate and manage self-destructive thoughts (72) and find rest (46). Stillness practices which cultivated self-awareness helped people find inner healing by connecting with their inner truth and re-envision their lives (77, 80).

#### 3.3.4 Emotional processing

Identifying, feeling, and moving through trauma-related emotions (e.g., grief, loss, laughter, learned helplessness, isolation, guilt, anger, sadness, and shame), rather than blocking them out, was instrumental in facilitating healing in many interventions (69, 74, 82). Moreover, processing emotions in a community-oriented, cultural context led to a sense of catharsis where a burden had been lifted (1, 3, 63, 68, 74, 76, 83). Creative practices such as art, painting, and dance also led to perspectives and release of difficult emotions (64, 66). Embodied practices such as dance, mindfulness of body, and reflective walking were particularly helpful in energizing bodies, resolving tension, and discharging emotions (24, 45, 64, 65). Last, overtly spiritual practices such as sweat lodge added to the healing power of interventions by creating a space where participants could let go of past pains and reclaim spirit (85).

#### 3.3.5 Raising consciousness

Consciousness raising in the forms of education and truth telling sparked healing among participants. For many, understanding history and the influence of societal factors (e.g., colonization, genocide) on present conditions (e.g., violence against Indigenous women, substance use) helped people accept and acknowledge the past, put present experience into context, and move forward (13, 22, 68, 73, 85). Sometimes, just learning the language about historical trauma helped honor experiences that had always been felt but rarely named and validated (13, 20, 81). Acts of truth telling, whether in story circle or through art-making, was healing for it allowed participants to understand and share about the ongoing effects of historical trauma such as mental health challenges and racism, realizing ultimately that they are not alone in their experiences (21, 76).

#### 3.3.6 Changing narratives

Healing also occurred as people changed their individual and collective stories. This is powerfully demonstrated in the case of a Navajo teenager whose suicidal ideation decreased significantly when she was able to separate her personal responsibility from collective responsibility (71). Individual narratives were changed through artistic practices which helped people see their lives in an alternative light (82) and reclaim their voice (15, 84). Often, narratives changed through resisting and challenging dominant narratives such as degrading representations, colonized land, capitalistic labor, and collective silence (13, 15, 23, 46, 68). As participants continued to see their stories reflected in the stories of others within a group, they built a sense of shared identity which fostered collective transformation (20, 82, 83, 85).

#### 3.3.7 Growing empowerment

The last form of healing occurred through a found sense of empowerment. Oftentimes, this arose as participants connected with the resilience coming from their inner strength (65, 77, 80) as well their traditional and spiritual practices (22, 77, 79, 81). Empowerment also arose from learning new skills such as reading, writing, making money by selling crafts, and learning positive life skills (67, 81, 82). As empowerment grew, it fostered a sense of hope, appreciation, and optimism for their lives, leading to a greater self-efficacy to enact changes in their personal lives and for their community (13, 24, 64, 73, 83).

## 4 Discussion

This study presented a scoping review of contemplative-based interventions for healing historical trauma among BIPOC communities across the globe. Publication trends demonstrate a steadily growing interest in this topic, particularly within the last decade, which may further reflect increasing overlapping interests in historical trauma and contemplative research. Of the 36 interventions identified across 35 articles, almost all the interventions were group- or community-based, reflecting the importance of collective healing for people who come from communities affected by systemic oppression and violence (86). Oftentimes, historical violence disconnects one from one's own culture and sense of community. Research with group or community-level interventions, such as macro-therapeutic interventions (87) and community healing models, may help to foster individual and collective identity and align more broadly with collective cultures. Based on the structure and duration of intervention designs, weekly sessions or intensive workshops may be most suitable for group- and community-level interventions.

As a whole, the results indicate a growing presence of contemplative intervention work that strongly centers on Indigenous populations in the United States, Australia, and Canada. Interventions integrated contemplative practice across the full spectrum of intervention levels and settings. The most common contemplative practices were creative, ritual, stillness, or relational, suggesting that future intervention design may focus on providing a combination of these practices. Considering that most interventions implemented a wide variety of practices, both contemplative and non-contemplative, having a variety of available practices might be helpful to enact multiple mechanisms of healing for participants.

A novel finding from this scoping review was the high prevalence of cultural practices among the interventions, many of which related to Indigenous practices and ceremonies that are grounded in spiritual beliefs and understandings. The prevalence of interventions focused on cultural revitalization may be influenced by the representation of Indigenous samples, for whom cultures have been targeted for erasure by settler colonialism. Globally, Indigenous communities have been practicing their own traditions to survive and heal from enduring legacies of historical trauma for centuries (88, 89). These traditions include ceremonies, wisdom practices, applied cultural knowledge, and collective care, many of which involve aspects of contemplation. Indigenous contemplative practices are grounded in collective well-being, relationality, and connections to land, culture, and spirituality. Unlike many Western models of secular mindfulness that emphasize contemplative practices at an individual level (90), these practices are embedded within community and ancestral knowledge systems. The literature highlights ceremony and prayer, storytelling, and land-based practices as key approaches that support reflection, healing, and intergenerational knowledge transmission. Additional practices include song, drumming, dance, Elders' teachings, and circle-based exercises, all of which emphasize embodied reflection, listening, and communal healing.

Collectively, these culturally grounded contemplative practices are framed as supporting Indigenous resilience, identity, and healing across generations, and should be recognized more explicitly in the contemplative science literature as effective, evidence-based approaches for collective healing. Of all articles, only a few made direct linkages between contemplation and Indigenous paradigms (69, 72, 84). This finding points to a gap in the literature where Indigenous practices and ceremonies, many of which are deeply contemplative in nature (30), are not considered contemplative practice due to a lack of communication between the major fields of contemplation research and historical trauma. Similar gaps in connecting culturally-specific practices to contemplative practices likely exist among racial and ethnic minorities represented in this sample of literature, such as scarf dance in Korean culture (65), spiritual practices among Hmong communities (13, 91), and African spiritual wisdom (47). Due to the small number of articles for other racial and ethnic minorities, an extended exploration of this gap is beyond the scope of the manuscript. Nevertheless, integrating these bodies of literature for BIPOC communities at large is imperative for elevating intrinsic healing contemplative practices within communities and culturally adapting contemplative interventions to better support long-lasting improved health outcomes related to historical trauma.

### 4.1 Healing mechanisms

This study identified seven processes related to healing that involve establishing safety, cultural revitalization, increasing well-being, emotional processing, raising consciousness, changing narratives, and growing empowerment. The findings align with literature in the contemplative field that has documented the benefits of contemplative practices for supporting emotional regulation (92, 93), promoting interpersonal connection (94), and establishing grounded and authentic spaces of healing (44, 50). The healing mechanisms also align with French et al.'s (97) radical healing paradigm for people of color, noting that raising critical consciousness of the impact of past oppressions on one's current experience is crucial for healing to occur.

While the findings reflect the clear presence of radical healing among the interventions, many of the articles did not focus specifically on the role of contemplative practices in healing. Therefore, the mechanisms presented represent a broad understanding of healing mechanisms across interventions and only mention the role of contemplative practice if it was directly noted in the article. The contemplative practices most directly noted included creative practices, stillness practices, relational practices, and spiritual practices. An unexpectedly limited number of studies focused specifically on embodiment in facilitating healing, though some studies did specifically note the role of accessing and integrating or releasing stored traumas in the body and the power of leaning into discomfort for emotional processing (45, 65). This gap may indicate that, while the theoretical foundations connecting historical trauma and embodiment are supported, targeting embodiment is only one of many pathways to reintegrate trauma in practice. Research on trauma-informed mindfulness has shown that targeting somatic reintegration of internalized traumas through contemplative practice can cause further harm if not led in trauma-informed ways (41). Furthermore, this finding may be due to the study designs represented, which are limited in their measurement of embodiment as a primary outcome. The high prevalence of creative healing mechanisms suggests that the overall process of healing from historical trauma is overall a creative one (86), highlighting the important role that art can play in redefining individual and collective narratives.

### 4.2 Study design and methodology

The scoping review identified a rich variety of methods used due to the variation in types of intervention settings, intervention aims, and sample characteristics. In terms of study design, the prevalence of non-experimental one-arm pilots reflects both the relative recency of research interventions for healing historical trauma as well as the need to develop and adapt interventions suited for each community given the uniqueness of each community's history and present needs. The recency of this literature is ironic given the longstanding traditions from which culturally grounded contemplative practice arises; therefore, the trend is not a reflection of the history of the practices themselves but of the growing recognition of these practices within research paradigms and their adaption into established social work agencies.

Based on their majority of representation among study methodologies, qualitative and mixed methods studies are well-suited for understanding the impact of such designed interventions, ongoing evaluations, and other interventions arising out of community organizing. In turn, these methods are also suitable to the emergent nature of scientific exploration of the phenomenon of contemplative healing of historical trauma. Qualitative research is well-suited for gaining nuanced understanding of participant experiences given how the effects of historical trauma are myriad and interlinked, as are the mechanisms of healing. Quantitative surveys proved helpful in cases where there were specific outcomes connected to historical trauma such as substance use, suicide, grief, and trauma symptoms; but no measures were found that directly measured historical trauma symptoms. Far fewer scales have been developed specifically for measuring historical trauma. The Historical Trauma and Associated Symptoms Scale (95) was developed for use within Indigenous populations and has since been adapted for other populations such as Southeast Asians (96). Given the specificity of impact within each community, further use of scales for measuring historical trauma among other communities would best be developed specific to each community's historical and social context.

As this field grows, the evidence base may be strengthened with more integration of experimental designs and long-term follow-up, particularly to replicate findings of pilot studies. The results included an unexpectedly high number of case reports based on existing programs and community-organized events. Furthermore, full-text review excluded numerous high-potential contemplative interventions which did not meet criteria for counting as intervention studies. These included writing poetry, collective organizing, meditative practices, theatrical enactments of history, and hip-hop music. This study defined intervention studies as those which implemented a specific program with traditional methods for gathering data. While this criteria helped to establish rigor in study selection, widening the definition of intervention research may include a wider scope of interventions that can lead to positive, meaningful outcomes for community, thereby expanding the praxis of future research on this topic.

### 4.3 Community engagement

An important consideration for future research on contemplative interventions for healing is the community-based aspect of intervention development and research design. All but one intervention (71) was facilitated either by a community insider or outsider with significant experience working with the community and related issues. Establishing safety is a tenant of trauma-informed practice, reflected in the results as a requisite for healing to occur. Given the history of extraction, violence, and alienation experienced by many of these communities, the establishment of safety through community partnerships in research and intervention development is crucial.

However, Community-Based Participatory Research (CBPR) is a term that can be overly used. Based on Key et al. (57)'s Continuum of Community Engaged Research, studies qualify as CBPR if the community being studied is meaningfully involved throughout the entire process and retain a sense of ownership over the work. For instance, one article described using CBPR but provides little to no description of efforts to include people from the community or people that have worked extensively with the community in the research team (76). It was also not explicitly stated in much of the research that the data or outcomes would be owned by or returned to the communities that were researched. These oversights are concerning when working with Indigenous and diasporic communities that have been historically exploited, as they can reinforce distrust and cause further harm. When engaging with these communities in further research in this area of contemplative healing from historical trauma, research teams committed to CBPR must involve community from the very beginning and sustain this partnership throughout the research process to build mutuality and collaboration, especially considering that many contemplative practices are culturally embedded.

### 4.4 Limitations and future directions

This study has several additional limitations that are imperative to discuss. First, search terms related to contemplative practice and trauma healing could have been expanded to identify further relevant studies. The possible overrepresentation of certain geographic regions and populations may be related to the used search terms and could be addressed by future studies with a wider array of search terms. Second, most studies did not describe interventions in considerable detail. For instance, creative practices in particular were mentioned most often as simply “painting,” “art,” or “singing,” which poses difficulties in replicating studies and understanding the specific impact of their intervention components. It is likely that having no embedded quality assessment, another limitation of the study, contributed to the lack of specificity in defining intervention components across numerous studies. Future studies would benefit from having clearer definitions of interventions as part of a quality assessment. Finally, having a single primary reviewer conducting screening and coding introduced potential bias in study selection, extraction, and interpretation of findings, such as in the categorization of contemplative practices. For instance, many Indigenous spiritual ceremonies and rituals likely have generative components, but because they were not explicitly described in the articles themselves, they were not included in the generative practice category. This limitation was addressed by having regular research meetings with subject matter experts, and also by including Indigenous scholars who could give critical feedback on the writing team.

### 4.5 Conclusion

Despite these considerations, this scoping review offers a foundation for future research to explore healing mechanisms within specific branches of contemplative practice, such as relational, stillness, creative, and spiritual practices, in relation to historical trauma. Subsequent work can incorporate a broader and more inclusive range of community-identified interventions and deepen understanding of how these practices may support healing among BIPOC communities affected by historical trauma. Additional research is also needed to explore the implementation of contemplative approaches within communities in ways that maintain cultural integrity while remaining adaptable and replicable cross-culturally.

The findings presented here point to the importance of future interdisciplinary research that documents intervention details, investigates healing mechanisms across contemplative branches, and tests implementation models that are both community-driven and adaptable. This approach will strengthen the design of contemplative interventions that honor Indigenous epistemologies, support embodied and collective healing, and advance equitable public health responses to historical trauma. The fields of contemplative practices and historical trauma can be advanced effectively by bridging Indigenous studies and BIPOC community practice with contemplative science, so long as this work continues to be carried out in deep partnership with and for BIPOC communities.

## Data Availability

The original contributions presented in the study are included in the article/supplementary material, further inquiries can be directed to the corresponding author.
